# Influence of Root Diameter and Soil Depth on the Xylem Anatomy of Fine- to Medium-Sized Roots of Mature Beech Trees in the Top- and Subsoil

**DOI:** 10.3389/fpls.2017.01194

**Published:** 2017-07-24

**Authors:** Kristina Kirfel, Christoph Leuschner, Dietrich Hertel, Bernhard Schuldt

**Affiliations:** Plant Ecology, Albrecht von Haller Institute for Plant Sciences, University of Göttingen Göttingen, Germany

**Keywords:** cambial aging, deep roots, *Fagus sylvatica*, hydraulic conductivity, high-conductivity roots, vascular differentiation, vessel diameter

## Abstract

Despite their importance for water uptake and transport, the xylem anatomical and hydraulic properties of tree roots have only rarely been studied in the field. We measured mean vessel diameter (*D*), vessel density (VD), relative vessel lumen area (lumen area per xylem area) and derived potential hydraulic conductivity (*K*_p_) in the xylem of 197 fine- to medium-diameter roots (1–10 mm) in the topsoil and subsoil (0–200 cm) of a mature European beech forest on sandy soil for examining the influence of root diameter and soil depth on xylem anatomical and derived hydraulic traits. All anatomical and functional traits showed strong dependence on root diameter and thus root age but no significant relation to soil depth. Averaged over topsoil and deep soil and variable flow path lengths in the roots, *D* increased linearly with root diameter from ∼50 μm in the smallest diameter class (1–2 mm) to ∼70 μm in 6–7 mm roots (corresponding to a mean root age of ∼12 years), but remained invariant in roots >7 mm. *D* never exceeded ∼82 μm in the 1–10 mm roots, probably in order to control the risk of frost- or drought-induced cavitation. This pattern was overlain by a high variability in xylem anatomy among similar-sized roots with *K*_p_ showing a higher variance component within than between root diameter classes. With 8% of the roots exceeding average *K*_p_ in their diameter class by 50–700%, we obtained evidence of the existence of ‘high-conductivity roots’ indicating functional differentiation among similar-sized roots. We conclude that the hydraulic properties of small to medium diameter roots of beech are mainly determined by root age, rendering root diameter a suitable predictor of hydraulic functioning, while soil depth – without referring to path length – had a negligible effect.

## Introduction

Water uptake and transport is a key function of the root system and essential for plant growth and survival. Despite their decisive role for the provision of water to the shoot, the anatomical and hydraulic properties of the root system have only rarely been studied in comparison to the hydraulic system of aboveground organs (e.g., Brunner et al., 2015). This particularly applies to deep roots (McElrone et al., 2004; Gebauer and Volařík, 2013; Maeght et al., 2013), although their importance for water uptake especially in dry periods is well recognized (Stone and Kalisz, 1991; Domec et al., 2004; Bleby et al., 2010; David et al., 2013).

The function of a tree’s hydraulic system is largely determined by the number, diameter, and length of xylem conduits within the network of conducting elements from roots to leaves (Lintunen and Kalliokoski, 2010; Schuldt et al., 2013; Kotowska et al., 2015). Different xylem anatomical designs represent functional adaptations to variation in water availability among other environmental factors (Tyree et al., 1994a). High hydraulic conductance facilitates high rates of water movement and tree growth, but may imply high vulnerability to cavitation and xylem dysfunction induced by frost and drought (Tyree, 2003a; Hajek et al., 2014). The hydraulic architecture of trees therefore results from a trade-off between mechanical requirements, hydraulic safety, and hydraulic efficiency, with the latter being most effectively provided by large conduit diameters according to the Hagen–Poiseuille law, since increases in conduit diameter exponentially enhance hydraulic conductivity (Tyree and Zimmermann, 2002).

The anatomy of the xylem is highly heterogeneous at the interspecific level but also within a species or even a single tree (Sperry and Saliendra, 1994; Sperry et al., 2006; Lachenbruch et al., 2011; Schuldt et al., 2013; Chenlemuge et al., 2015; Kotowska et al., 2015). As one general structural principle of the hydraulic architecture of trees, a pattern of radial variation in xylem anatomy and hence in hydraulic performance from pith to bark has frequently been observed in the stems of both angiosperm and gymnosperm trees: in general, the density of tracheids and vessels decreases in radial direction, while conduit length and diameter increase (Gartner, 1995; Tyree, 2003b; Christensen-Dalsgaard et al., 2008; Fan et al., 2009). This radial gradient in anatomical structure is thought to be caused by the process of cambial aging, and is generated, depending on the species, most pronouncedly in the first 5 to 40 years of a tree organ’s lifespan (Fan et al., 2009; Lachenbruch et al., 2011). However, addressing cambial maturation does not provide a mechanistic explanation and solely describes a developmental process that results in a change in the dimensions of cambial initials over time, which in turn affects the dimensions of xylem cells produced by the cambium (Spicer and Gartner, 2001).

While the phenomenon of basipetal and radial conduit widening has often been recognized in tree stems, studies investigating radial patterns of xylem anatomy in tree roots are scarce. For the roots of Douglas-fir trees, the pattern could partly be confirmed by one study (Peterson et al., 2007), while Dunham et al. (2007), in contrast, reported a decrease in tracheid diameter and length, and specific conductivity with cambial age. Lintunen and Kalliokoski (2010) observed a generally large intra- and interspecific variation in radial xylem anatomical patterns in the roots of three different tree species. Opposite to a trend toward smaller but more numerous conduits from pith to bark in the roots of *Pinus sylvestris*, in the roots of *Betula pendula* and *Picea abies*, conduit frequency decreased and mean conduit size increased from the pith to the bark in agreement with observations at the stem base.

Apart from such radial gradients of root hydraulic architecture, most studies recognized a successive increase in the diameter of xylem conduits from the terminal branches to the stem, and further to the roots (Aloni, 1987; Tyree and Zimmermann, 2002; Hacke et al., 2016).

Soil depth-dependent changes in xylem architecture and hydraulic performance of roots have as well only rarely been the object of scientific study (e.g., Gebauer and Volařík, 2013; Maeght et al., 2013; Wang et al., 2015; Pierret et al., 2016). The results of the few existing studies indicate a gradient in root axial hydraulic conductivity as a result of xylem anatomical adaptations with increasing soil depth (Tyree, 2003b). McElrone et al. (2004) reported decreasing conduit radii from the most distant 20 m deep-reaching roots to the shallow surface roots, and to the stem wood in four tree species of different systematic position and growth habit (evergreen vs. deciduous, angiosperm vs. gymnosperm). Correspondingly, Pate et al. (1995) observed a progressive increase in mean xylem conduit diameter and specific hydraulic conductivity (30- to 150-fold) from the stem to the lateral roots and with soil depth in the sinker roots of different Proteaceae species. Investigating xylem anatomical and hydraulic properties in small roots of two different oak species at different soil depths, Gebauer and Volařík (2013) found a higher specific hydraulic conductivity due to larger vessel diameters in roots in 50 cm depth than at the surface, but no further increase in these traits from 50 to 100 cm depth. They assumed that vessel diameters in roots at the soil surface are limited in order to avoid cavitation due to freeze-thaw cycles. In three temperate hardwood tree species, a depth-dependent increase in specific hydraulic conductivity was observed in the tree fine root system: first-order roots exhibited 78 to 217% greater specific hydraulic conductivities in the subsurface (20–30 cm soil depth) than in the surface layer (0–10 cm soil depth) in the same species (Wang et al., 2015). In this case, the higher hydraulic efficiency appeared to be not solely a function of wider maximum conduit diameters, but also to result from a higher conduit frequency and greater xylem to cross-sectional area ratio.

Systematic influences of position in the conductive system and cambial age on the xylem anatomy and hydraulic architecture of roots may be masked by a great anatomical variation across roots of the same soil depth and age (Rewald et al., 2011; Köcher et al., 2012; Hajek et al., 2014). These authors observed an anatomically deviating form of roots which they termed ‘high-conductivity roots’ with an up to 10-fold higher specific hydraulic conductivity compared to the mean of roots. Such specialized roots were found in mature trees of several deciduous species including *Fagus sylvatica*. The high hydraulic conductivity of these roots was in most cases caused by the existence of a few very large vessels, but in others also by a large increase in vessel density, or by a combination of both anatomical adaptations. It is not well understood how frequent such ‘high-conductivity roots’ are and in which part of the root system they are occurring.

In this study, we analyzed the intraspecific variability in xylem anatomical and derived hydraulic traits of small- and medium-sized roots (1–10 mm in diameter) in the top- and subsoil down to a depth of 200 cm in a mature *F. sylvatica* L. (European beech) forest stand in Northern Germany. We hypothesized that (i) vessel diameter and hydraulic conductivity is a function of root diameter and, thus, of root age, (ii) the variability in xylem anatomical and hydraulic traits in similar-sized roots is high at a given soil depth with some roots exhibiting characteristics of ‘high-conductivity roots,’ and (iii) vessel diameter and consequently hydraulic conductivity increase with increasing soil depth.

## Materials and Methods

### Study Site and Field Sampling

The study site is located in the Grinderwald in the Pleistocene lowlands of Lower Saxony, Germany, 33 km northwest of Hannover (52° 34′ 22,115 North, 9° 18′ 49,762 East), 106 m above sea level. The climate is cool-temperate with a mean annual temperature of 8.7°C, and a mean annual precipitation of 718 mm. The even-aged mature forest stand was established in 1914 and is dominated by *F. sylvatica* L. with admixture of single trees of other species. Mean stem density is 407 stems ha^-1^, mean diameter at breast height is 26.3 cm, and mean basal area 27.1 m^2^ ha^-1^. The predominant soil type at the study site is an acid (pH 3.4–4.5), sandy Dystric Cambisol which developed from Pleistocene fluvial and aeolian sandy deposits from the penultimate (Saale) glaciation.

In order to analyze the soil depth influence on the wood anatomical and derived hydraulic properties of the roots, fine-, small- and medium-sized (Supplementary Table S1) beech root segments were collected in autumn 2013 in three soil pits that were dug to 200 cm depth in the stand. Root segments of ∼10 cm length originating from the neighboring trees were sampled on the 200 cm-wide profile walls at 7 soil depths from 0–20 to 160–200 cm. In each soil pit and soil depth, 6–10 root segments were selected covering all root diameters between 1 and 10 mm, yielding 197 analyzed root segments in total (Supplementary Table S1). The sampled root segments were cleaned from soil residues and immediately transferred to 70% ethanol for storage.

Since we assumed a generally high variability in xylem anatomical and hydraulic traits for similar-sized roots, we additionally investigated the effect of root age on root xylem characteristics and hydraulic properties in individual root strands for being able to separate age effects on root anatomy from possible depth-dependent and flow-path length induced changes in these traits. Therefore, we additionally excavated four complete root strands (root individuals with their main axes and appending secondary and higher-order branch roots) belonging to three different tree individuals located in the organic and topsoil layer. From each strand, 6–10 segments covering as many root diameter classes between 2 and 10 mm as possible were processed, yielding 42 analyzed segments in total.

### Xylem Anatomical and Derived Hydraulic Properties of Beech Roots

All root samples were stained with safranin (1% in 50% ethanol, Merck, Darmstadt, Germany) and washed with 70% ethanol prior to cutting. Subsequently, 10–20 μm semi-thin transverse sections were cut using a sliding microtome (G.S.L.1, WSL Birmensdorf, Switzerland). We processed and analyzed images of each cross-sectional transverse section taken with a stereo-microscope equipped with an automatic stage and a digital camera (SteREOV20, Carl Zeiss MicroImaging GmbH, Göttingen, Germany) at 100× magnification using Adobe Photoshop CS6 (version 13.0 x 64, Adobe Systems Incorporated, United States) and the particle analysis function from ImageJ (version 1.49 v). Root age (years) was determined by counting growth rings in each sample. However, contrary to stems and branches, growth rings in roots are sometimes difficult to identify and false rings might have been counted in certain roots. Consequently, our root age determination may partly over- or underestimate real age. The complete cross-section was analyzed (mean ± SE of analyzed root xylem area: 15.69 ± 0.95 mm^2^), yielding 116 to 5,871 measured vessels per sample, and 319,293 analyzed vessels in total. For the complete xylem cross-sectional area (*A*_xylem_, mm^2^) without bark, we determined vessel density (VD, n mm^-2^) and calculated the relative vessel lumen area (*A*_lumen_: *A*_xylem_, %), i.e., the relative proportion of cumulative vessel lumen area (*A*_lumen_, mm^2^) in percent of *A*_xylem_. The idealized mean vessel diameter (*D*, μm) was obtained from major (*a*) and minor (*b*) vessel radii according to the equation given by Lewis and Boose (1995) as $D={\left((32\times {\left(a\times b\right)}^{3})/\left(a^{2}+b^{2}\right)\right)}^{1_{{/}_{4}}}$. In addition to *D*, the maximum vessel diameter of a given root sample is given (*D*_max_, μm). The hydraulically weighted vessel diameter (*D*_h_, μm), in which each vessel is weighted proportionally to its contribution to total hydraulic conductance, was calculated from single vessel diameters (*D*) according to Sperry et al. (1994) as $D_{h}=\Sigma D^{5}/\Sigma D^{4}$. Potential hydraulic conductivity (*K*_p_, kg m^-1^ MPa^-1^ s^-1^) was calculated according to the Hagen–Poiseuille equation as $K_{p}=\left(((\pi \times \Sigma r^{4})/8\eta )\times \rho \right)/A_{xylem}$, where η is the viscosity of water (1.002 10^-9^ MPa s), ρ the density of water (998.2 kg m^-3^), both at 20°C, and *A*_xylem_ (m^2^) the corresponding xylem area.

### Statistical Analyses

Statistical analyses were performed with the software package R (R Core Team, 2013, version 3.4.0) except for linear regression analyses which were executed with the software XACT 8.03 (SciLab, Hamburg, Germany). During the analysis, normal distribution of the residuals and homogeneity of variance were assessed visually using residual diagnostics and quantile-quantile plots; if the assumption of normality was not met data were log-transformed. Linear mixed effect (LME) models with soil depth, root diameter and their interaction as fixed continuous variables were applied to analyze their influence on the xylem anatomical and derived hydraulic properties with the ‘*lme*’ routine of the ‘*nlme*’ package. We assumed non-independence of the three soil pits in the stand by adding soil pit as random effect. Additionally, we accounted for deviations from the assumed linear trend with soil depth resulting from spatial dependence in root samples by adding a random effect for a given soil depth as distinct variable nested in soil pit.

In order to estimate the variability in xylem anatomical and hydraulic traits in similar-sized roots, we divided the dataset into 9 different root diameter classes (1–2, 2–3 mm, and so on). The ratio of diameter class variance component to total variance was calculated using the R package ‘*varComp*’ according to a variance component analysis with the program ‘*lme*’ to calculate the proportion of total variance explained by the variability between root diameter classes $\left(\sigma_{\mathrm{int}er}^{2}\right)$ and residual variance within root diameter classes $\left(\sigma_{\mathrm{int}ra}^{2}\right)$. Variance component between root diameter classes (VC_inter_) was calculated according to ${VC}_{\mathrm{int}er}=(\sigma_{\mathrm{int}er}^{2}/\left(\sigma_{\mathrm{int}er}^{2}+\sigma_{\mathrm{int}ra}^{2}\right))\times 100$ and variance component within root diameter classes (VC_intra_) as ${VC}_{\mathrm{int}ra}=(\sigma_{\mathrm{int}ra}^{2}/\left(\sigma_{\mathrm{int}ra}^{2}+\sigma_{\mathrm{int}er}^{2}\right))\times 100$, all in percentage. We additionally calculated VC_inter_ and VC_intra_ for the variability between and within the seven investigated soil depth classes.

## Results

### Effect of Root Diameter on Xylem Anatomical and Derived Hydraulic Traits

**FIGURE 1:**
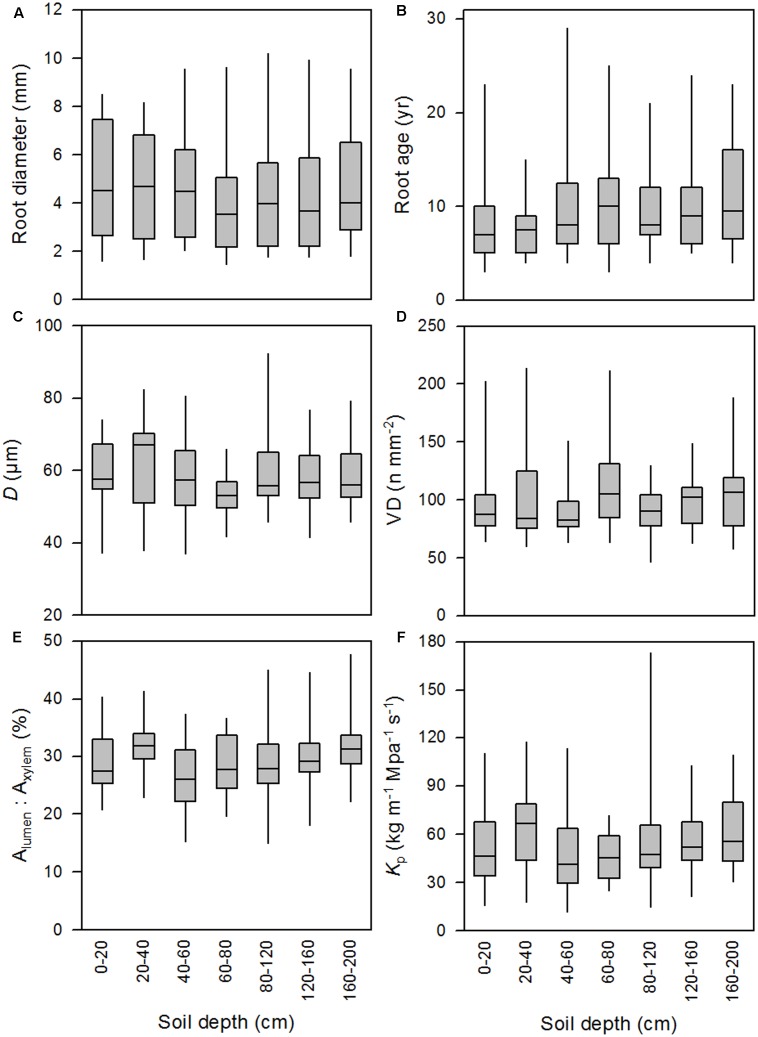
Box-whisker plots (with median, 25 and 75% quantiles and extreme values) for the variation in root diameter **(A)**, root age **(B)**, mean vessel diameter (**C**, *D*), vessel density (**D**, VD), relative vessel lumen area (**E**, *A*_lumen_: *A*_xylem_), and potential hydraulic conductivity (**F**, *K*_p_) in seven different soil depth classes.

**FIGURE 2:**
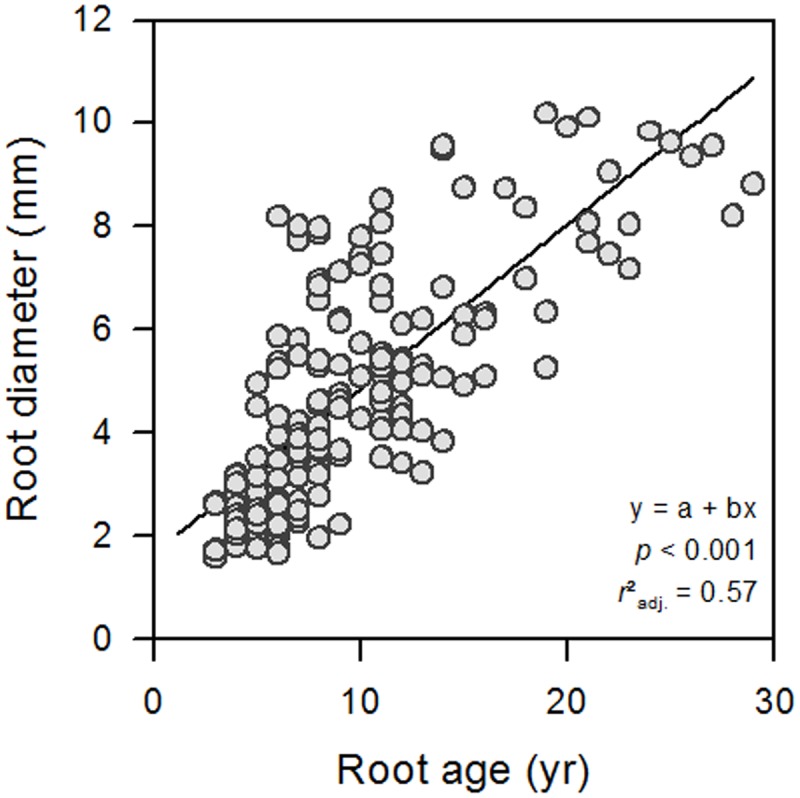
Root diameter in relation to root age in the sample of 197 roots.

**FIGURE 3:**
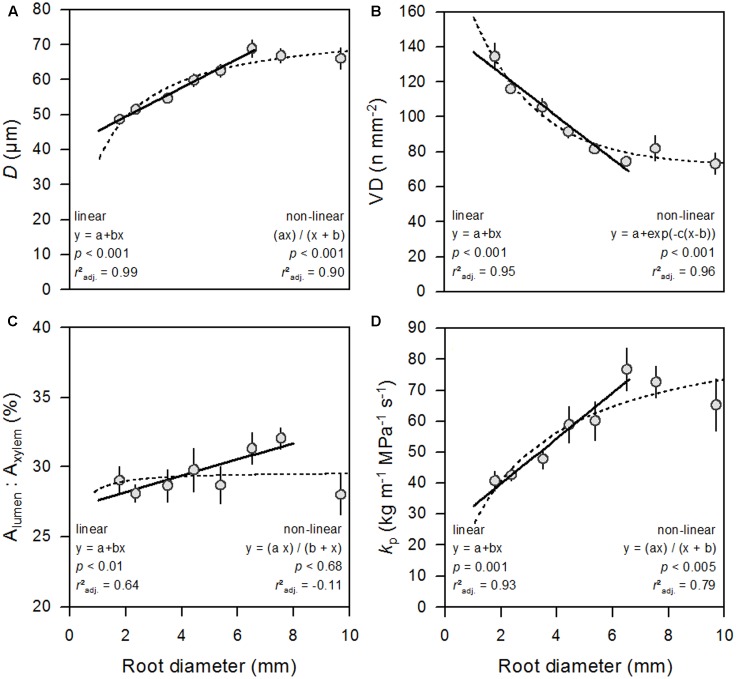
Root diameter in relation to mean vessel diameter (**A**, *D*), vessel density (**B**, VD), relative vessel lumen area (**C**, *A*_lumen_: *A*_xylem_), and potential hydraulic conductivity (**D**, *K*_p_). Values are means ±1 SE.

The results of the LME model covering roots of all diameter classes from all seven soil depth classes down to a depth of 200 cm (see Supplementary Table S1 and **Figure 1A**) showed a significant influence of root diameter on all studied xylem anatomical and hydraulic traits (**Table 1**). Although root diameter varied considerably among the roots of a given root age, both parameters were tightly linked to each other (*P* < 0.001; *r*^2^ = 0.57; **Figure 2**). The regression analyses exhibited a strong linear positive relationship between root diameter and mean vessel diameter (*D*) and potential hydraulic conductivity (*K*_p_) up to the diameter class 6–7 mm, followed by a slight decrease in *D* (from ∼69 to ∼66 μm) and *K*_p_ for larger roots (**Figures 3A,D**). The mean *K*_p_ values were 42.6, 59.0 and 60.2 kg m^-1^ MPa^-1^ s^-1^ for roots of 2–3, 4–5, and 5–6 mm in diameter, respectively (**Figure 3D**). Inversely, mean vessel density (VD) significantly declined with increasing root diameter up to the diameter class 6–7 mm (**Figure 3B**). Since the hydraulically weighted vessel diameter (*D*_h_) exhibited the same relationships to the analyzed parameters as *D*, we refrain from discussing this parameter further in order to avoid redundancies.

**Table 1:** Results of linear mixed effects models on the influence of soil depth and root diameter as fixed continuous variables on eight wood anatomical variables in roots of European beech (*Fagus sylvatica*) (*n* = 197), and the interaction of both factors.

	Soil depth			Root diameter			Soil depth:root diameter			VC_intra_	VC_inter_	VC_intra_	VC_inter_
					
Variable	Δi	LR	*P*	Δi	LR	*P*	Δi	LR	*P*	Root diameter class		Soil depth class	
Age	0.27	2.27	0.13	96.86	98.86	**<0.001**	1.82	0.18	0.67	31.40	68.60	96.08	3.92
*A*_xylem_	1.18	0.82	0.37	406.85	408.85	**<0.001**	0.57	2.57	0.11	5.27	94.73	99.53	0.47
*A*_lumen_:*A*_xylem_	1.24	3.24	0.07	6.40	8.40	**0.004**	2.22	4.22	**0.04**	98.46	1.54	91.10	8.90
VD	1.55	0.45	0.50	64.95	66.95	**<0.001**	1.70	0.30	0.58	51.38	48.62	97.52	2.48
*D*	1.97	0.03	0.86	53.44	55.44	**<0.001**	1.92	0.08	0.78	50.35	49.65	96.13	3.87
*D*_max_	0.13	1.87	0.17	77.39	79.39	**<0.001**	1.68	0.32	0.57	45.04	54.96	97.43	2.57
*D*_h_	1.35	0.65	0.42	36.46	38.46	**<0.001**	1.98	0.02	0.90	62.92	37.08	98.24	1.76
*K*_p_	0.61	2.61	0.11	27.59	29.59	**<0.001**	0.53	1.47	0.23	74.95	25.05	95.88	4.12


*Studied traits are root age (years), cross-sectional xylem area (*A*_*xylem*_, *mm^2^*), relative vessel lumen area (*A*_*lumen*_: *A*_*xylem*_, %), vessel density (*VD, n mm^-2^*), vessel diameter (*D*, *μm*), maximal vessel diameter (*D*_*max*_, *μm*), hydraulically weighted vessel diameter (*D*_*h*_, *μm*) and potential hydraulic conductivity (*K*_*p*_, *kg m^-1^ MPa^-1^ s^-1^*). Also expressed is the variation of the traits (variance component, VC in %) within (*VC*_*intra*_) and between (*VC*_*inter*_) different root diameter classes (*n* = 9) and soil depth classes (*n* = 7). Given are the delta Akaike information criterion (Δi), the likelihood ratio (LR) and probability of error (*P*-value). Significant correlations (*p* < 0.05) are shown in bold.*

Despite the significant correlation between root diameter and xylem anatomical and hydraulic parameters, we found a high variability in these traits for similar-sized roots. Within a root diameter class, *D* varied between the extremes by 54–80% and *K*_p_ by 210–720% (average variation of *D* and *K*_p_ by ∼39 and ∼76%, respectively). Accordingly, the variance component of both traits within a diameter class is similar to, or larger than that between diameter classes (**Table 1**).

### Existence of ‘High-Conductivity Roots’

**FIGURE 4:**
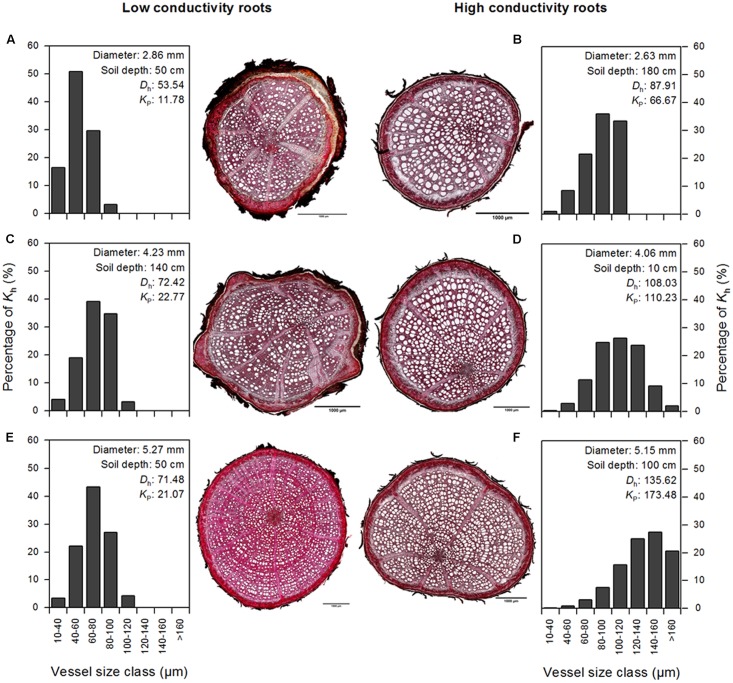
Cross-sections at 100× magnification for three pairs of roots of each similar diameter (**A,B**: 2.7 mm; **C,D**: 4.1 mm; **E,F**: 5.2 mm) displaying typical ‘low conductivity’ (left) and ‘high conductivity’ (right) characteristics, and relative contribution of eight vessel size classes to theoretical hydraulic conductivity (*K*_h_). The depicted scale bars represent 1 mm.

In our sample of 197 studied roots, we found 16 roots with a large number of vessels >100 μm diameter and therefore particularly high axial conductivity. We termed roots with at least 50% higher *K*_p_ values than the average of its diameter class ‘high-conductivity roots’ and roots with only 50% or less of average *K*_p_ ‘low-conductivity roots.’ **Figure 4** presents microscopic pictures of the anatomy and the vessel size distribution for pairs of high- and low-conductivity roots in three different root diameter classes. The existence of roots with particularly high potential hydraulic conductivity appears to be independent of soil depth. Unlike similar-sized roots with lower *K*_p_ values, which typically are characterized by left-skewed vessel size distributions, high-conductivity roots possess a large proportion of vessels with medium or large diameters and tend more to a right-skewed distribution. In analogy to the dependence of vessel size and hydraulic conductivity on root diameter in the whole data set (**Figure 3**), mean *D* and *K*_p_ of high-conductivity roots linearly increased to a maximum value at root diameters of 6–7 or 5–6 mm, respectively, and then leveled off in roots of larger diameters (data not shown). In the high-conductivity roots of this beech stand, mean *D* did not exceed a value of ∼82 μm.

### Effect of Soil Depth on Xylem Anatomical and Derived Hydraulic Traits

The results of the LME model revealed no significant influence of soil depth on the studied xylem anatomical and hydraulic characteristics (**Table 1**). Even when the relationship is analyzed separately within the nine root diameter classes, no clear pattern of a soil depth influence on *D* emerged although vessels declined in size with depth in the root diameter class 4–5 mm, but they increased in size in the root diameter class 9–10 mm (Supplementary Figure S1). Likewise, maximum vessel diameter (*D*_max_) did not increase with soil depth but remained more or less unaltered around 126.15 ± 1.75 μm (mean ± SE, *n* = 7) across the vertical profile (Supplementary Figure S2).

Similarly, comparison of the studied root traits in different soil depth classes did not show significant vertical gradients in *D* VD, *K*_p_, root age and relative vessel lumen area (*A*_lumen_:*A*_xylem_) (**Figures 1B–F**). Accordingly, the variance component of all variables tested was larger within a given soil depth class than that between depth classes (**Table 1**).

### Detailed Analysis of Individual Root Strands

**FIGURE 5:**
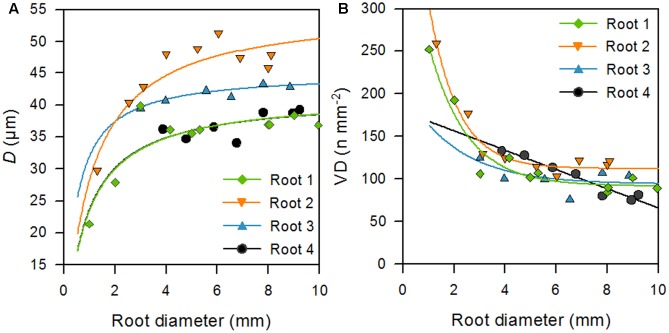
Root diameter of single root strands in relation to mean vessel diameter (**A**, *D*) and vessel density (**B**, VD). Regression functions, adjusted coefficients of determination (*r*^2^_adj._) and probability of error (*P*-value) are given in **Table 2**.

In order to investigate the diameter dependence of the seven studied root traits independent of possible path-length induced or depth-dependent changes in these variables, we conducted a detailed study in four selected root strands from the topsoil, complementing our main analysis of 197 segments from a large number of roots. In three of the four roots, *D* was hyperbolically related to root diameter and turned to an asymptote at root diameters of 6–8 mm, never exceeding maximum *D*-values of ∼51 μm (**Figure 5A** and **Table 2**). This corresponds to the results of the regression analysis between root and vessel diameter in the main analysis (**Figure 3**). This pattern was not found in root #4, probably because small-diameter segments with most rapid vessel diameter increase were not present in this strand. This root further showed a linear, and not an exponential, decrease of VD with increasing root diameter, contrary to the pattern observed in root #1 and #2 (**Figure 5B** and **Table 2**).

**Table 2:** Results of regression analyses between the diameter of single root strands and the corresponding mean vessel diameter (*D*, μm), vessel density (VD, n mm^-2^) (shown in **Figure 5**) and potential hydraulic conductivity (*K*_p_, kg m^-1^ MPa^-1^ s^-1^) at the cross-section.

Variable	Unit	Root no.	Regression function	*r*^2^_adj._	*P*
*D*	μm	1	y = (41.5130^∗^x)/(x + 0.7698)	0.76	**<0.001**
		2	y = (55.2948^∗^x)/(x + 0.9587)	0.83	**<0.001**
		3	y = (45.0381^∗^x)/(x + 0.4087)	0.80	**<0.050**
		4	y = (45.0381^∗^x)/(x + 0.4087)	0.26	0.135
VD	n mm^-2^	1	y = (91.3833 + exp(-0.6727(x - 8.5868))	0.93	**<0.001**
		2	y = 111.9291 + exp (-0.8945 (x - 6.8872))	0.95	**<0.001**
		3	y = 93.7398 + exp (-0.4910 (x - 9.6527))	-0.07	0.284
		4	y = 178.6284 - 11.2725x	0.93	**<0.001**
*K*p	kg m^-1^ MPa^-1^ s^-1^	1	y = 5.2304 + 0.1774x	-0.04	0.219
		2	y = (37.1319^∗^x)/(x + 2.5028)	0.53	**<0.050**
		3	y = 8.1632 + 0.5034x	0.08	0.149
		4	y = 8.5599 - 0.2249x	0.01	0.178


*Given are the number of observed segments along the individual root, the adjusted coefficient of determination (*r*^2^_adj._), and the probability of error (*P*-value). Significant correlations (*p* < 0.05) are shown in bold.*

## Discussion

### Effects of Root Diameter and Root Age on Wood Anatomical and Hydraulic Properties

In agreement with our first hypothesis, we could confirm that mean vessel diameter (*D*) and potential hydraulic conductivity (*K*_p_) in beech roots are a function of root diameter and thus of root age. We found *D* and *K*_p_, analogously, to increase linearly from the root tip in proximal direction to a maximum of ∼70 μm and ∼77 kg m^-1^ MPa^-1^ s^-1^, respectively, in medium-sized roots at a diameter of 6–7 mm, corresponding to a mean root age of ∼12 years. In thicker roots, *D* remained constant at this vessel diameter level while *K*_p_ decreased to a level of ∼65 kg m^-1^ MPa^-1^ s^-1^ in the root diameter class from 8 to 10 mm. The inverse pattern was found for the relationship between root diameter and vessel density (VD). The longitudinal diameter-dependent patterns in *D* and VD were characteristic for the majority of investigated root strands and were also present in the ‘high-conductivity roots.’ However, *D* remained constant at a value of ∼82 μm in the latter and already at ∼51 μm in the other (‘normal’ or low-conductivity) root strands. This observation may suggest that maximum vessel diameter is restricted already in medium-sized beech roots (∅ 5–10 mm), perhaps for avoiding drought- or frost-induced cavitation. Larger conduit sizes greatly increase the risk of freeze-thaw (Mayr et al., 2006; Pittermann and Sperry, 2006; Christensen-Dalsgaard and Tyree, 2014) and drought-induced embolism (Hargrave et al., 1994; Tyree et al., 1994a,b; Hajek et al., 2014), presumably because wider vessels may have thinner and more porous pit membranes compared to narrower ones (Hacke et al., 2016). Already 30 years ago, Tyree and Sperry (1989) speculated that the development of frost- or drought-induced embolism is not directly influenced by conduit size but rather indirectly by pit properties, a hypothesis recently confirmed by Li et al. (2016). The authors identified pit membrane thickness as key determinant of embolism resistance across a broad range of woody angiosperm species. However, independent of the mechanisms underlying the increased risk of cavitation in wider vessels, maximum vessel size may well be limited to balance all of these requirements, since conduit size is thought to display a trade-off between hydraulic efficiency and safety, as well as mechanical requirements.

As a general structural principle of the hydraulic architecture of trees, it is assumed that conduit diameters increase with increasing distance from the apex in order to maintain a constant flow rate along the entire path from the roots to the leaves (West et al., 1999; Zaehle, 2005). This architectural principle has been confirmed by a number of studies in the stems and branches of trees (e.g., Anfodillo et al., 2006; Petit et al., 2008; Petit and Anfodillo, 2009), but our knowledge on the belowground scaling of xylem conduits is very limited (e.g., Petit et al., 2009; Wang et al., 2015). For the long conducting lateral roots of dicotyledonous and monocotyledonous plants, Aloni (1987) suggested from a comprehensive review on vascular differentiation a universal pattern of continuous vessel diameter increase with increasing distance to the stem base. Petit et al. (2009) confirmed continuous tapering in the roots of small coniferous trees with increasing proximity to the stem base. Contrary to the findings of these authors, our observation of conduit widening with increasing diameter from small- to medium-sized roots seems to be at odds with the concept of conduit tapering which predicts that mean vessel size should decline along the flow path from the distal roots tips to the stem base as observed by Petit et al. (2009). While we do not know the path length of the studied root individuals, we can certainly infer that vessel diameters initially widen at least along the first part of the flow path from the root tip until the roots reach diameters of ∼7 mm in our investigated beech root sample. Vessel tapering may well occur, when roots grow thicker than 10 mm, but this was not studied here. Hence, the question whether conduit tapering is playing a similarly important role in the xylem of tree root systems as it seems to do in the crown remains unanswered at this point.

In support of our first hypothesis, our results prove that vessel diameter increases with increasing root diameter and thus with root age, suggesting, that in this root diameter range, vessel diameter is predictable by organ diameter. Similarly, a vessel diameter–stem diameter relation has been observed by various other authors (e.g., Coomes et al., 2007; Olson and Rosell, 2013; Olson et al., 2014; Pfautsch, 2016; Rosell et al., 2017). This finding matches the frequent observation of a radial increase in conduit diameter from the pith to the bark in the stem of angiosperm and gymnosperm trees (Gartner, 1995; Lachenbruch et al., 2011). However, information on radial variation in root xylem anatomical and hydraulic properties is yet very limited. Several studies separating root branching orders showed that conduit diameter tends to increase toward higher root orders, in agreement with our findings (Valenzuela-Estrada et al., 2008; Huang et al., 2010; Long et al., 2013; Gu et al., 2014), while others, however, failed to detect a radial conduit widening from the pith to the bark in roots (Dunham et al., 2007; Peterson et al., 2007; Lintunen and Kalliokoski, 2010). This may suggest that the radial pattern of xylem anatomy in woody roots is often overlain by adaptive responses to locally varying mechanical requirements (Christensen-Dalsgaard et al., 2008).

#### Drivers of Vessel Development

Gradual radial change in xylem anatomy is commonly assigned to the process of cambial maturation, addressing the aging of cambial initial cells over time, which is thought to cause changes in the dimension of xylem cells formed (Spicer and Gartner, 2001). In the first years of growth when trees produce juvenile wood, the cambial initials undergo rapid change and the size of conduits formed typically increases. After 5–40 years, depending on species, the increase in conduit diameter levels off and mature wood with more uniform xylem anatomical properties is produced (Spicer and Gartner, 2001; Mäkinen et al., 2007; Christensen-Dalsgaard et al., 2008; Fan et al., 2009). This gradual increase in conduit diameter exponentially increases hydraulic efficiency because flow in capillary systems increases with diameter raised to the fourth power according to the Hagen–Poiseuille law. However, the mechanisms underlying the process of cambial maturation are not well understood. Some evidence suggests that age-related differences in xylem differentiation result from a complex interplay between plant hormones, gene expression, and environmental influences (Li et al., 2012). It is well established that the plant hormone auxin – in concert with further plant hormones such as gibberellins, cytokinins and ethylene – is a key regulator in plant vascular development (Nilsson et al., 2008; Aloni, 2015). Auxin is synthesized in the developing leaves, resulting in a longitudinal gradient in auxin concentration along the flow path from the apex to the roots (Aloni and Zimmermann, 1983; Uggla et al., 1998; Aloni, 2015; Hacke et al., 2016; Pfautsch, 2016). However, auxins are also synthesized in the roots with concentrations decreasing from the tip to more proximal root sections, as shown for *Arabidopsis* (Ljung et al., 2005; Teale et al., 2006; Petersson et al., 2009). Although it is not fully understood how auxin modulates vessel size patterns (Teale et al., 2006; Anfodillo et al., 2012), its involvement in turgor-driven cell growth suggests that auxin plays a role in determining vessel diameters (Hacke et al., 2016). Frequent observations of vertical vessel widening from the apex to the stem base, and radially from the pith to the cambium, have led to the hypothesis that gradients in auxin concentration are responsible for this vascular modification with flow path length or cambial age (Aloni and Zimmermann, 1983; Lachenbruch et al., 2011; Anfodillo et al., 2013). Applied to our data, a decreasing auxin concentration with distance from the root tip in parallel to the observed larger vessel diameters with increasing root diameters could well explain the vessel widening with increasing root diameter. However, a consistent relationship between auxin concentration and variation in xylem differentiation has not always been demonstrated (Zajaczkowski, 1973; Sundberg et al., 1993; Little and Pharis, 1995; Uggla et al., 1998). While it is undisputed that auxin is a key regulator in secondary xylem development, differences in the responsiveness of cambial cells to different auxin levels may be a cause of variation in xylem anatomical traits (Nilsson et al., 2008).

Investigations on the molecular level offer clues on the mechanisms underlying the phenomena ascribed to cambial maturation suggesting that xylem differentiation differs with age in dependence of ontogenetic change in gene expression (Lenz et al., 2010; Li et al., 2012). Comparing the regulation of xylem candidate genes at different tree ages, Li et al. (2010) observed that many of the relevant genes are preferentially expressed in certain development phases or tree ages, resulting in variations in transcript abundance at different stages of cambial maturity. For example, the expression of cell wall related genes generally decreased with cambial age. It is evident that our understanding of genetic regulation of cambial aging is still rudimentary and needs further intensive study (Li et al., 2010).

### Variability in Xylem Anatomical and Hydraulic Traits among Similar-Sized Roots

Another main result of this study is the high plasticity in xylem anatomy and related hydraulic properties in beech roots of similar size co-occurring at the same soil depth. Despite the relative scarcity of information about xylem anatomical and hydraulic properties of tree roots, a growing number of studies provide evidence of a generally high heterogeneity in these traits, as evidenced in the studies of, e.g., Leuschner et al. (2004) and Rewald (2008) for temperate hardwoods. This suggests that morphologically and anatomically different roots in the same soil horizon may also serve different functions, for example predominantly nutrient absorption, or alternatively water uptake and conduction. Functional specialization may develop in response to gradients in nutrient and water availability, as has been indicated by a number of studies (Pierret et al., 2007; Rewald et al., 2011; Köcher et al., 2012; Hajek et al., 2014).

Thus, the observed high variability in the wood anatomical and derived hydraulic properties of the beech roots in this soil is presumably a consequence of the considerable heterogeneity in soil texture and soil water content at the site (Supplementary Table S2; John et al., under reveiw). We speculate that high-conductivity roots have contact to soil patches where water is, or was, more easily available stimulating vessel diameter growth, in contrast to other roots which may predominantly be responsible for nutrient uptake (Pierret et al., 2007). In general, the marked anatomical and functional plasticity in secondary vascular elements is considered to be of high adaptive significance in response to climatic conditions and other external factors (Carlquist, 2001; Spicer and Groover, 2010). Alternatively, the wide vessels in high-conductivity roots could also result from greater pathway length, i.e., longer distal fine root strands than in the average of roots in that diameter class. The higher conductivity of the more proximal root segment would then simply balance the higher cumulative resistance in the longer distal flow path.

We found ‘high-conductivity roots’ in six of seven soil depth classes between the surface and 200 cm depth. Seventy-five percent of these roots were found in the subsoil below 60 cm depth. We observed ‘high-conductivity roots’ in all studied diameter classes from fine to medium roots (1–10 mm in diameter). Their particularly high hydraulic conductivity is mainly a result of larger mean vessel diameters and not higher vessel density. This is in accordance with conclusions from earlier work that conduit diameter is the main determinant of axial conductivity in roots (McElrone et al., 2004; Köcher et al., 2012; Gebauer and Volařík, 2013; Hajek et al., 2014), since an increase in conduit radius scales the flow in capillary systems exponentially by a fourth-power relationship (Tyree and Zimmermann, 2002). In comparison, the plasticity of the branch hydraulic system seems to be lower in temperate broad-leaved trees: vessel size distribution was found to be more balanced than in roots with no indication of ‘high-conductivity branches’ (Hajek et al., 2014).

### Influence of Soil Depth on Xylem Anatomical and Related Hydraulic Properties

Our study did not produce evidence for increases in vessel diameter and potential hydraulic conductivity with increasing soil depth in mature beech trees. In contrast to our findings, Pate et al. (1995) found a progressive increase in mean xylem conduit diameter and specific hydraulic conductivity down to 1.3 m depth for sinker roots of various Proteaceae species. Likewise, McElrone et al. (2004) observed increases in mean *D* and hydraulic efficiency from shallow to deep roots (7–20 m soil depth) for several tree species.

Several factors may be responsible for the apparent discrepancy between the reported soil-depth effects on xylem anatomy and our results. First, it may be that the depth-dependent gradients in xylem anatomical and hydraulic traits reported in the cited studies are reflecting differences in path length between the investigated root sections, while we were not able to measure root length and thus path length to the stem base and the related position of the segment in the flow path. This is the reason why we refer to soil depth, and this could explain differences to the above-mentioned studies.

Second, the few existing studies on conduit scaling in roots indicate that the rooting system is much more responsive to external factors (Nardini et al., 2002; Christensen-Dalsgaard et al., 2008) like freeze-thaw events (Gebauer and Volařík, 2013) or water availability (Rewald et al., 2011; Köcher et al., 2012), and mechanical demands (Dunham et al., 2007; Lintunen and Kalliokoski, 2010) than the crown. Gebauer and Volařík (2013) found an increase in mean vessel diameter and specific hydraulic conductivity from 0 to 50 cm in the roots of two temperate oak species, but no further scaling in these traits from 50 to 100 cm soil depth, concluding that vessel size in the upper soil layer is restricted in order to avoid freeze-thaw-induced embolism formation. In contrast to McElrone et al. (2004); Lintunen and Kalliokoski (2010) reported a tendency for sinker roots to have smaller conduit radii than shallow roots of the same species, concluding that hydraulic conductivity of sinker roots might be traded off against the mechanical demand of anchoring the tree firmly to the soil under conditions of ample moistures.

## Conclusion

We found key anatomical and hydraulic traits of beech roots to scale with root diameter in roots of ≤7 mm in diameter, pointing at a dominant root age effect on belowground hydraulic properties. In the first months to years of their life beech roots grow in diameter to 6–7 mm and *D* increases to a threshold value of 70–80 μm. As a result, the capacity for water conduction doubles and activity shifts from resource uptake to transport and storage functions. The threshold *D* level may be defined by safety requirements to avoid embolism.

From the observed large variability in anatomical properties among similar-sized neighboring roots it is evident that the age-related pattern is overlain by a high xylem architectural plasticity of the root system. This heterogeneity might either be attributable to spatial variation in the influence of external factors or to differences in flow path length from the distal root to the stem base. It appears that different functional types of roots with respect to water uptake and conduction do exist in the root system of beech trees, which deserve more detailed study.

To separate between the influential factors, future studies on root vascular anatomy adjustment in soil profiles should account for path length effects in the root strands. This would require excavating larger parts of the tree root system instead of sampling individual root sections only, and thus is very labor-intensive and destructive, when done in mature forests.

## Author Contributions

KK and BS: Substantial contributions to the conception/design work, the acquisition, analysis, and interpretation of data for the work; Drafting the work; Final approval of the version to be published; Agreement to be accountable for all aspects of the work in ensuring that questions related to the accuracy or integrity of any part of the work are appropriately investigated and resolved. CL and DH: Substantial contributions to the conception/design of the work; Revising the work critically for important intellectual content; Final approval of the version to be published; Agreement to be accountable for all aspects of the work in ensuring that questions related to the accuracy or integrity of any part of the work are appropriately investigated and resolved.

## Conflict of Interest Statement

The authors declare that the research was conducted in the absence of any commercial or financial relationships that could be construed as a potential conflict of interest.
