# Supplementary material for: Influence of Root Diameter and Soil Depth on the Xylem Anatomy of Fine- to Medium-Sized Roots of Mature Beech Trees in the Top- and Subsoil
Source: Front Plant Sci. 2017 Jul 24;8:1194. doi: 10.3389/fpls.2017.01194 (PMC5522885; doi:10.3389/fpls.2017.01194)
Supplement: Supplementary file 1 [file Data_Sheet_1.pdf]

Supplementary Material

Influence of root diameter and soil depth on the xylem anatomy of fine- to medium-sized roots of mature beech trees in the top- and subsoil

Kristina Kirfel, Christoph Leuschner, Dietrich Hertel, Bernhard Schuldt*

*Correspondence: bernhard.schuldt@plant-ecology.de

Table S1: Root classification according to diameter after Sutton and Tinus (1983) and number of observations (n) per root class and soil depth (cm) across the three excavated soil pits.

**Root diameter**	**Classification**	**Soil depth (cm)**						
		**0 - 20**	**20 - 40**	**40- 60**	**60 - 80**	**80 - 120**	**120 - 160**	**160 - 200**
Ø 1-5 mm	fine and small roots	16	15	15	19	18	18	19
Ø 5-10 mm	medium roots	14	11	13	7	10	11	11
Ø 1-2 mm		2	4	0	4	4	5	1
Ø 2-3 mm		7	3	9	6	9	6	7
Ø 3-4 mm		3	3	4	7	3	4	7
Ø 4-5 mm		4	5	2	2	4	3	4
Ø 5-6 mm		4	1	5	5	4	4	2
Ø 6-7 mm		1	5	4	0	2	1	3
Ø 7-8 mm		4	4	0	1	1	0	3
Ø 8-9 mm		5	1	2	0	0	3	1
Ø 9-10 mm		0	0	2	1	1	3	2

Table S2: Physical and chemical soil characteristics at different soil depths in the Grinderwald forest (June 2013). Classification of soil horizons according to FAO - WRB 2014.

Soil depth (cm)	Soil horizon	pH ($CaCl_2$)	SOC ($g\ kg^{-1}$)	Sand (%)	Silt (%)	Clay (%)
0-2	AE	3.3	27.0	70.0	26.0	4.0
2-12	Bsw	3.4	17.0	65.0	30.0	5.0
12-36	Bw	4.4	7.0	67.0	29.0	4.0
36-65	BwC	4.5	3.0	73.0	24.0	3.0
65-125	C	4.4	0.4	95.0	4.0	1.0
125-150	2C	4.1	0.1	81.0	11.0	8.0
150-180	2Cg	4.2	0.8	72.0	19.0	9.0
180+	3C	4.2	<0.1	95.0	4.0	1.0

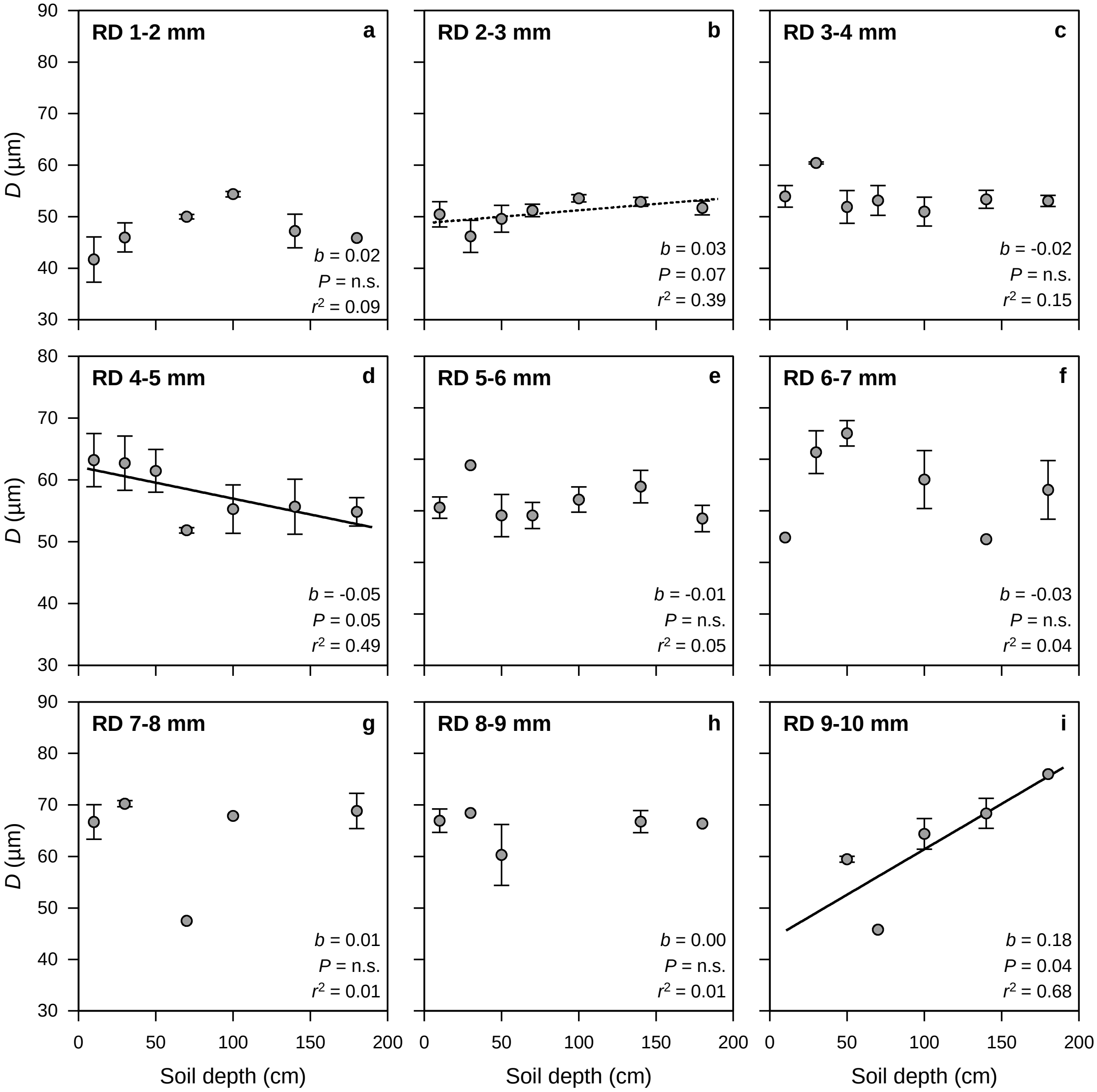

Figure S1: Influence of soil depth on mean vessel diameter (*D*) for nine different root diameter classes (RD). For each root diameter class, 9-44 samples were available, which subsequently were averaged for each soil depth class. For number of replicates per root diameter class see Table S1. Values are means ± SE; the slope (*b*), coefficient of determination (r^2) and probability of error (*P*-value) of the linear regressions are given.

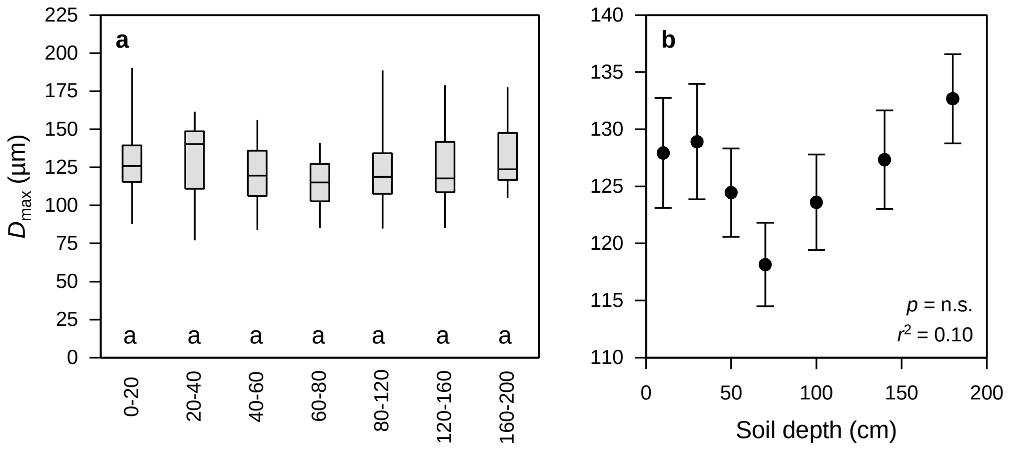

Figure S2: Box-whisker plots (with median, 25 and 75% quantiles and extreme values) for the variation in maximum vessel diameter (D_{max}) in seven soil depth classes (a); small letters indicate significant differences between depth classes. Additionally given is the relation between soil depth and mean values ± SE for D_{max} (b). Please not the different scaling of the y-axis.
